# Integrated genome-wide association and transcriptomic studies reveal genetic architecture of bulb storability of plentiful garlic germplasm resources

**DOI:** 10.1093/hr/uhae260

**Published:** 2024-09-16

**Authors:** Yue Zhu, Huixia Jia, Jiangping Song, Tingting Zhang, Xiaohui Zhang, Wenlong Yang, Yumin Tan, Mengzhen Wang, Jiyan Zang, Haiping Wang

**Affiliations:** State Key Laboratory of Vegetable Biobreeding, Institute of Vegetables and Flowers, Chinese Academy of Agricultural Sciences, 12 Zhongguancun South Street, Haidian District, Beijing 100081, China; State Key Laboratory of Vegetable Biobreeding, Institute of Vegetables and Flowers, Chinese Academy of Agricultural Sciences, 12 Zhongguancun South Street, Haidian District, Beijing 100081, China; State Key Laboratory of Vegetable Biobreeding, Institute of Vegetables and Flowers, Chinese Academy of Agricultural Sciences, 12 Zhongguancun South Street, Haidian District, Beijing 100081, China; State Key Laboratory of Vegetable Biobreeding, Institute of Vegetables and Flowers, Chinese Academy of Agricultural Sciences, 12 Zhongguancun South Street, Haidian District, Beijing 100081, China; State Key Laboratory of Vegetable Biobreeding, Institute of Vegetables and Flowers, Chinese Academy of Agricultural Sciences, 12 Zhongguancun South Street, Haidian District, Beijing 100081, China; State Key Laboratory of Vegetable Biobreeding, Institute of Vegetables and Flowers, Chinese Academy of Agricultural Sciences, 12 Zhongguancun South Street, Haidian District, Beijing 100081, China; State Key Laboratory of Vegetable Biobreeding, Institute of Vegetables and Flowers, Chinese Academy of Agricultural Sciences, 12 Zhongguancun South Street, Haidian District, Beijing 100081, China; State Key Laboratory of Vegetable Biobreeding, Institute of Vegetables and Flowers, Chinese Academy of Agricultural Sciences, 12 Zhongguancun South Street, Haidian District, Beijing 100081, China

## Abstract

Garlic is a widely utilized condiment and health product. However, garlic bulbs are prone to quality deterioration resulting in decrease of economic value during postharvest. In this study, the storability of 501 garlic accessions worldwide was evaluated based on the examination of decay index (DI), decay rate, sprouting rate, and bud-to-clove ratio in two consecutive years. The DI was employed as a primary index for evaluating the storability of garlic. Among these garlic, 43 accessions exhibited strong storability with DI of 0%–5%. Phenotypic and cytological observations revealed that strong storability accessions displayed delayed sprouting and decay, a slow rate of nutrient transfer to vascular bundles. Through genome-wide association study (GWAS), 234 single-nucleotide polymorphism loci (SNPs) were associated with the storability, which were located in or near 401 genes, which were annotated the functions of resistance, storage substances transport, etc. A total of 44 genes were screened using selective sweep analysis. Transcriptomic analysis was performed at four periods after storage in the 8N035 accession with strong storability and 8N258 accession with weak storability. Compared with 8N035, the upregulated genes in the 8N258 were enriched in photosynthesis and stress response, whereas the downregulated genes were enriched in response of biotic and abiotic stress and defense response. A co-expression network and GWAS identified three hub genes as key regulatory genes. Conjoint analysis of GWAS, selective sweep, and transcriptomic analysis identified 21 important candidate genes. These findings provided excellent resources with storability and vital candidate genes regulating storability for biological breeding of garlic.

## Introduction

Garlic (*Allium sativum* L*.*) is an important vegetable and medicinal plant. Aside from being a natural spice with unique flavor [1], it is an excellent dietary source of nutrients and phytochemicals because of its richness in alliin, polysaccharides, and other active compounds. Besides, garlic is also utilized to formulate drugs for treating illnesses and as preservatives [2–4]. In 2022, garlic production is 34 million tons worldwide (FAOSTAT). As the biggest garlic exporter, China accounts for 80% of the international garlic trade.

Garlic has a long growth cycle, up to 7–8 months, so it can only plant one growth period per year. To maintain a shelf life throughout the year, its primary product organ, bulbs, needs to be stored well. During postharvest storage, garlic bulbs will experience physiological disorders, sprouting and decay, leading to a significant decrease in quality and economic value [5–7]. In normal circumstance, the amount of loss incurred while in storage can be up to 40% [8]. Currently, this loss can be reduced through improving storage environment. Extensive examination of aspects such as temperature, humidity, air composition, and packaging materials has been used to formulate corresponding industry standards [9–13]. Nevertheless, controlling the storage environment requires a lot of labor and cold storage space, which implies high costs. In order to fundamentally solve the problem of storage loss, it is essential to find excellent germplasm or develop new varieties with strong storability.

Several researches in garlic storage process have been performed from the nutritional ingredients, hormones, bacteria, etc. During storage, sprouting of cloves is the main factor affecting the storage quality. After sprouting, the levels of carbohydrates, anthocyanins, and carotenoids decrease, while peroxidase and gibberellin A_3_ (GA_3_) increase [14, 15]. Abscisic acid, which is antagonistic to GA_3_, has been utilized as an external inhibitor of sprouting. In addition, a high concentration of bacteria and viruses in garlic bulbs greatly increases the possibility of rot. *Fusarium oxysporum f.* sp. cepae (FOC) is the main pathogen responsible for this rot [16]. It has been determined that garlic has certain genes, like *pathogenesis-related* (*PR*), *Thaumatin-like Protein* (*TLP*), *dehydration responsive element binding* (*DREB*), and *sugars will eventually be exported transporter* (*SWEET*) family member, that are related to *Fusarium* resistance [17–20]. Revealing the regulatory mechanism of quality decline during storage is beneficial to improve the quality of garlic after long-term storage. However, this mechanism has not been fully elucidated in the present studies.

Garlic is an asexually propagated crop, so its traditional breeding mainly relies on physical and chemical mutations, which are inefficient and non-directional for breeding. The utilization of modern molecular breeding technology, such as molecular markers, genetic transformation, and gene editing, can greatly improve the breeding efficiency for asexually propagated crops [21–23]. To apply molecular breeding technology into garlic breeding, it is necessary to find the key loci or genes that control the target traits. Thus, it is important to elucidate the genetic architecture of garlic bulb storability and screen key regulatory genes and candidate genes to accelerate the process of molecular breeding.

**Fig. 1:**
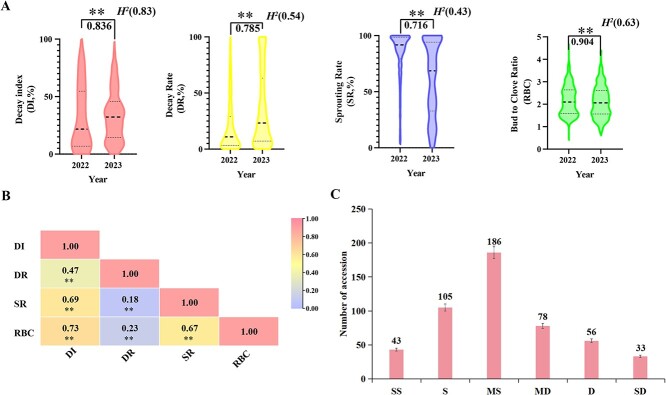
Statistical analysis of four storability-related traits of garlic bulbs. **(A)** Violin diagram in two consecutive years. The thick dashed line is the average, and the thin dashed line is the quartile. The values below the line are the correlation coefficients between two consecutive years (Pearson’s r). ^**^ means highly significant correlation at the 0.05 level. *H^2^* is the broad-sense heritability. **(B)** Heat map of correlation coefficients among the four traits. **(C)** Number of garlic accessions into six groups: SS (0%–5%), S (5%–15%), MS (15%–40%), MD (40%–60%), D (60%–75%), SD (75%–100%).

Genome-wide association study (GWAS) is a powerful technique for studying the connection between phenotype and genotype. Integrated GWAS with transcriptional regulation analysis can enable the identification of key regulatory genes. This approach has been successful in finding genes that regulate quality and disease resistance in a variety of food and vegetable crops [24–26]. Following the publication of the garlic reference genome [27], it has been extensively employed in the study of garlic yield and growth traits, thus providing a genetic basis and candidate genes for garlic breeding [28, 29]. Thus, the conjoint analysis of GWAS and transcriptome provides an efficient strategy for excavating the key regulatory genes for garlic bulb storability.

In this study, the storability of 501 garlic accessions from 38 countries was evaluated on a large-scale in two consecutive years. Phenotypic and cytological observations during storability of garlic bulbs were observed in detail. Combining GWAS and transcriptome studies, the genetic architecture and regulatory mechanisms of storability were revealed, and the candidate regulatory genes were identified. This study provides important germplasms with excellent storability and identifies essential candidate genes for molecular breeding in garlic production.

## Results

### Large-scale evaluation of storability of garlic bulbs

The storability of 501 garlic accessions from 38 countries (Table S1) was examined in two consecutive years by measuring the decay index (DI), decay rate (DR), sprouting rate (SR), and bud-to-clove ratio (RBC) of garlic bulbs. Analysis of correlation demonstrated that these four traits had highly significant correlation between two consecutive years, with correlation coefficients ranging from 0.716 to 0.904 (Fig. 1ATable S2). The DI was strongly correlated with the other three traits (Fig. 1B). Moreover, the heritability (*H^2^*) of the DI was 0.83, which was higher than the other three traits (Fig. 1A), suggesting that the DI was less influenced by environmental factors than the other traits. Therefore, the DI might be identified as a representative trait to evaluate the storability of garlic bulbs. Using the DI, 501 garlic accessions were separated into six groups: strong storability (SS) with DI of 0%–5%, storability (S) with DI of 5%–15%, medium storability (MS) with DI of 15%–40%, medium decay (MD) with DI of 40%–60%, decay (D) with DI of 60%–75%, and strong decay (SD) with DI of 75%–100%. A total of 43 accessions in SS group were identified as having strong storability, while 33 accessions in SD group were determined with high decay (Fig. 1C, Table S3).

### Morphological and cytological observations of garlic bulbs during storage

**Fig. 2:**
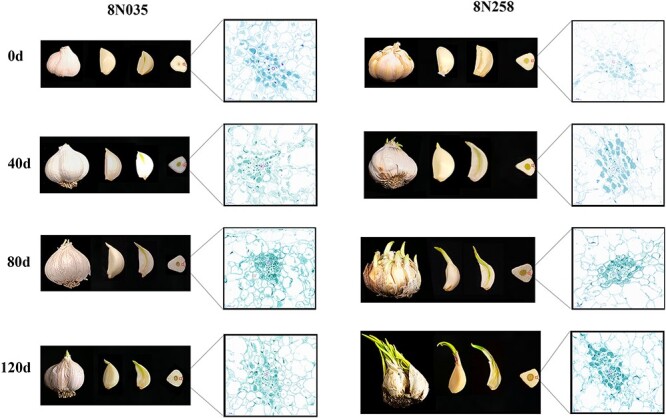
Morphological and cytological changes of garlic during storage. The changes of bulbs, cloves, cross and vertical section of cloves, and vascular bundle cells of the 8 N035 and 8 N258 accessions at 0, 40, 80, and 120 days after storage.

For in-depth analysis of the morphological and cytological changes, 8N035 accession in SS group and 8N258 accession in SD group were observed at 0, 40, 80, and 120 days after storage. During the early storage period (0–40 days), being broken of outer peels and sprouting of cloves were occurred in the 8N258, but were not in the 8N035. During the medium storage period (40–80 days), the outer peels of the 8N258 were completely broken, and all the cloves sprouted, while the outer peels and buds of the 8N035 were still intact. During the late storage period (80–120 days), the cloves of both the 8N035 and 8N258 began to turn yellow and shrink, but the buds of the 8N258 grew rapidly to >5 cm, which was significantly higher than the 8N035 with 2–3 cm. In the cytological observation of the vascular bundle on the back of cloves during storage, there was a clear phenomenon of gradual convergence of cellular contents toward the vascular bundles for bud growth in the 8N258. However, the same phenomenon was not apparent in the 8N035. In addition, in both 8N035 and 8N258, the cell around the vascular bundle parenchyma cell wall was observed to be broken, and this phenomenon was more severe in the 8N258 (Fig. 2).

**Fig. 3:**
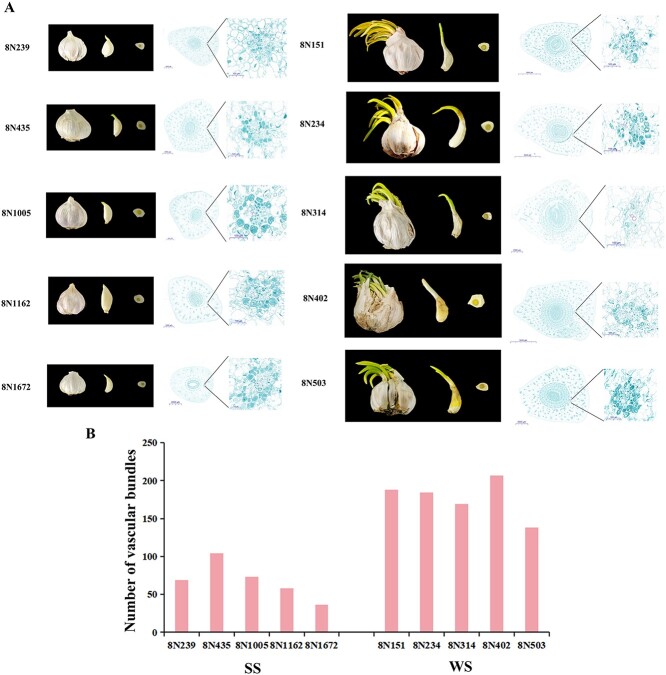
Morphological and cytological observation. **(A)** The difference of bulbs, cloves, cross-section in cloves, and vascular bundle cells of five accessions (8N239, 8N435, 8N1005, 8N162, 8N1672) with strong storability (SS) and five accessions (8N151, 8N234, 8N314, 8N402, 8N503) with weak storability (WS) at 120 days after storage. **(B)** The number of vascular bundles in each cross-section vertical section in cloves.

To further investigate the effect of vascular bundles on garlic storage, we selected other five accessions with strong storability (SS) and five accessions with weak storability (WS) for cytological observation after 120 days of storage. The results demonstrated that a greater proportion of cellular contents remained in the SS vascular cells than in the WS vascular cells. This observation was consistent with that made in 8N035 and 8N258. Furthermore, the distribution of vascular bundles in the cross-section of garlic cloves demonstrated that the number of vascular bundles was considerably higher in the WS accessions than in the SS accessions (Fig. 3). The enhanced number of vascular bundles and robust vascular transport capacity in WS accessions led to a greater quantity of cellular contents being transported from garlic cloves to buds. This might be an important reason for the severe quality loss of garlic cloves of the WS accessions.

### Genome-wide association and selective sweep analysis of storability-related traits

**Fig. 4:**
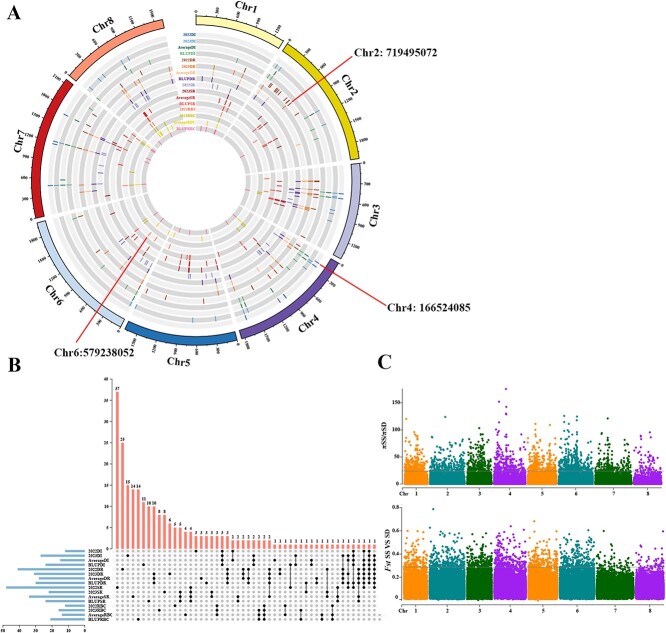
Genome-wide association and selective sweep analysis of storability-related traits. **(A)** Circos plot of garlic genome showing SNPs associated with the 2022 and 2023 data, average, and BLUP for four storage-related traits (DI, DR, SR, RBC). The Manhattan plots of all storage-related traits are shown in Fig. S1. **(B)** Upset Venn diagram showing the overlap number of the SNPs associated with the 2022 and 2023 data, average, and BLUP for four storage-related traits. The horizontal histogram illustrates the total number of SNPs in each data. The vertical histogram illustrates the number of SNPs associated with the data indicated by the black dots below. **(C)** Selective sweep analysis of *Fst* values and π ratio values between SS and SD accessions. The gray line represents the threshold for filtering the top 1% of *Fst* values (≥0.27) and πSS/πSD values (≥15.83) as the selected regions.

Four storability-related traits (DI, DR, SR, and RBC) were performed GWAS using mixed linear model (MLM). For each of these four storability-related trait, the 2022 data, 2023 data, average value and best linear unbiased prediction (BLUP) value for 2022 and 2023 were used for GWAS. When the -log_10_ (*P*-value) threshold was higher than 6.13, a total of 234 single-nucleotide polymorphism loci (SNPs) were screened out (Fig. 4A, B, Table S4). Notably, 27 SNPs were associated with the same trait in two consecutive years, including five SNPs (Chr3:472508318, Chr4:238163097, Chr4:1687871755, Chr7:475901220, Chr7:1212361479) associated with the DI, 13 SNPs (Chr1:1010132992, Chr3:261114114, Chr3:274424681, Chr3:439314285, Chr3:530026048, Chr3:826104883, Chr4:166524085, Chr4:637434589, Chr4:793765865, Chr6:26754872, Chr7:1212361479, Chr7:1551031522, Chr8:515280149) associated with the DR, four SNPs (Chr1:877914115, Chr1:877914200, Chr3:809664780, Chr5:794360243) associated with the SR, and five SNPs (Chr1:883248625, Chr7:390300047, Chr7:2050705189, Chr8:475421169, Chr8:565458301) associated with the RBC in 2022 and 2023 data (Fig. 4A, B, Table S4). In addition, the 13 SNPs were associated with two or more traits. Among these SNPs, five SNPs (Chr3:553898718, Chr4:166524085, Chr6:26754872, Chr7:475901220, Chr8:773483499) were repeatedly associated with DI, DR, and SR.

The genes associated with storability were identified through analyzing the associated SNPs in genic, intergenic, upstream, or downstream regions. A total of 401 candidate genes were identified according to the associated 234 SNPs (Table S4). Some of these candidate genes were annotated as encoding proteins related to stress resistance, defense mechanisms, and plant hormones (Table S5). For instance, the gene *Asa8G03362,* associated with RBC, is a transcription factor belonging to the MYB gene family, which has been found to be a key factor in flavonoid biosynthesis. Flavonoids could protect plant cells from the damage caused by reactive oxygen species (ROS) burst and play a role in defense against abiotic stresses [30]. *Asa6G00059* was located by Chr6:26754872, and encoded an F-box/LRR-repeat protein. Its homologous genes are involved in stress tolerance in soybean [31]. *Asa2G00638* and *Asa8G03714,* associated with 2022 DR, encoded DETOXIFICATION protein and ABC transporter G family member, respectively, and their homologous genes have been found to be related to drug resistance in other species [32, 33]. *Asa2G02374* that was associated with DR encoded alliinase, which reacted with alliin to produce allicin that was a strong antibacterial agent aside from being a main flavor component of garlic [2]. *Asa3G02513* that was associated with RBC encoded gibberellin 3-beta-dioxygenase. In addition, three non-synonymous SNPs (Chr2:719495072, Chr4:166524085, and Chr6:579238052) were located in the exonic regions of *Asa2G02643*, *Asa4G00601*, and *Asa6G02236*, respectively. *Asa2G02643* encoded pentatricopeptide repeat-containing protein At1g20230-like (Table S5), belonging to PPR protein family, and its homologous genes have profound effects on photosynthesis [34]. *Asa4G00601* encoded ribosomal protein S5 protein, which has been proven to be involved in protein synthesis and processing, and some extra ribosomal functions [35, 36]. *Asa6G02236* encoded autophagy-related protein 18a (Table S5), and its homologous gene attenuates plant resistance against necrotrophic pathogens in *Arabidopsis thaliana* [37].

To narrow down the associated regions involved in storability, the 43 SS accessions and 33 SD accessions were scanned for selective sweep analysis using *Fst* and πSS/πSD. The genomic regions with the top 1% cut-off (*Fst* ≥ 0.27, πSS/πSD ≥ 15.83, or πSS/πSD ≤ 0.31) were studied as potential regions under selection (Fig. 4C). A total of 44 genes were screened in potential regions and also found in GWAS result. Thereunto, several genes were annotated in relation to the transport of cellular contents. For example, *Asa3G03069*, *Asa4G00332*, and *Asa6G00058* were annotated as carbohydrate transport and metabolism. *Asa2G04659* was annotated as amino transport and metabolism. *Asa2G01090* and *Asa6G03998* were annotated as intracellular trafficking, secretion, and vesicular transport.

### Transcriptome sequencing and identification of differential expression genes

To examine transcriptional regulation mechanism and important regulatory genes that were involved in storability, the garlic bulbs of the 8N035 and 8N258 accessions at 0, 40, 80, and 120 days after storage were used for transcriptome sequencing, with three replicates for each sample set. A total of 307.36 Gb of clean data was gathered from 24 libraries, with a Q30 base percentage of >92.14%. Based on correlation and principal component analysis (PCA), the samples with three replicates had high correlation coefficients and cluster together, indicating the high-quality biological replicates (Fig. S2).

**Fig. 5:**
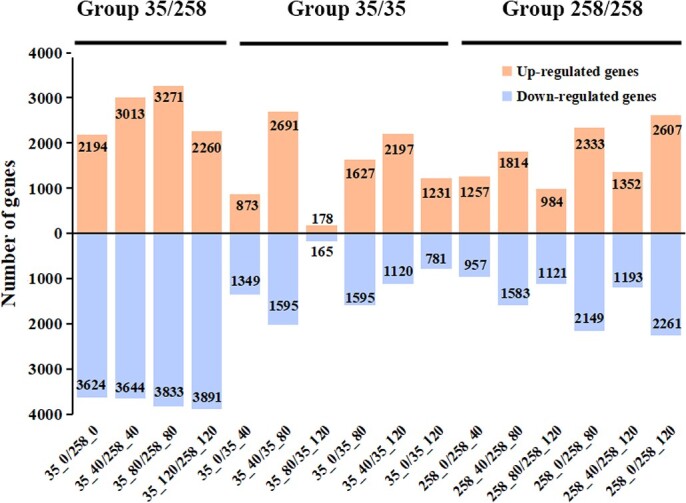
Quantity statistics of the DEGs between the 8N035 and 8N258 accessions and among four periods.

A total of 15,956 differentially expressed genes (DEGs) were identified by conducting 16 pairwise comparisons with the threshold of expression fold |Log_2_FoldChange| ≥ 1 and *Q*-value ≤.05. These 16 comparisons were divided into three groups: Group 35/258 comprised the comparison between the 8N035 and 8N258 accessions at the same storage period; Group 35/35 comprised the comparison among four storage periods in the 8N035 accession; Group 258/258 comprised the comparison among four storage periods in the 8N258 accession. The number of DEGs in Group 35/258 was significantly more than Group 35/35 and Group 258/258, being consistent with the obvious difference between the 8N035 and 8N258 accessions in the PCA plot (Fig. 5, Fig. S2). In Group 35/258, the number of upregulated genes increased gradually from 0 to 80 days, up to 3271 genes, and then began to decline. The number of downregulated genes increased gradually from 0 to 120 days, up to 3,891 genes. In Group 35/35 and Group 258/258, the upregulated genes had a similar trend with downregulated genes. In Group 35/35, the 35_40/35_80 comparison exhibited the greatest number of DEGs, with 2,691 upregulated and 2,024 downregulated genes. In 258/258, the 258_80/258_120 comparison yielded the largest number of DEGs, with 2,607 upregulated and 2,261 downregulated genes (Fig. 5, Fig. S3).

### Enrichment analysis of differential expression genes

**Fig. 6:**
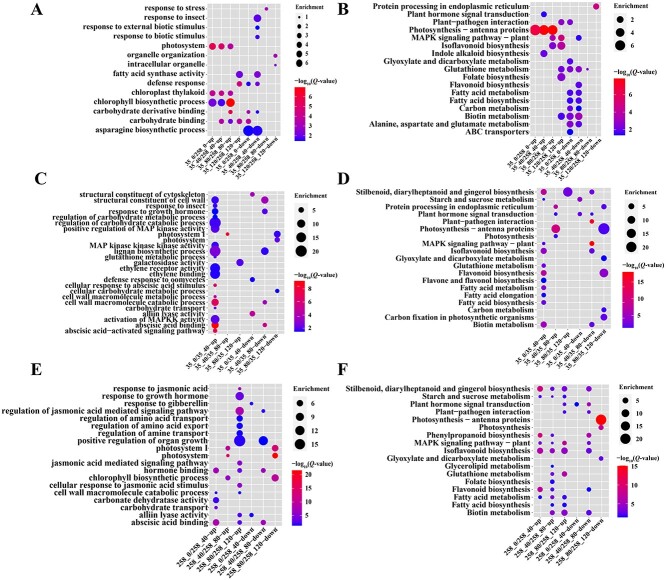
Enrichment analysis of the DEGs in the Group 35/258, 35/35, and 258/258. **(A)** GO enrichment analysis in Group 35/258. **(B)** KEGG enrichment analysis in Group 35/258. **(C)** GO enrichment analysis in Group 35/35. **(D)** KEGG enrichment analysis in Group 35/35. **(E)** GO enrichment analysis in Group 258/258. **(F)** KEGG enrichment analysis in Group 258/258.

The DEGs were functionally characterized through gene ontology (GO) and Kyoto Encyclopedia of Genes and Genomes (KEGG) enrichment analysis. Some of the terms and pathways about stress resistance, defense mechanisms, and plant hormones were enriched. Enrichment analysis of DEGs in the Group 35/258 was shown in Fig. 6A, B, and Table S6, S7. The upregulated genes mean the expression of these genes in 8N258 is higher than that in 8N035, while the downregulated genes mean the expression of these genes in 8N035 was higher than that in 8N258. After the 0-80 days storage, the upregulated genes were enriched in ‘chlorophyll metabolic and biosynthetic process’ and ‘photosystem and photosynthesis’, which could be linked to the growth of clove buds. Downregulated genes were enriched in substance biosynthesis and metabolism in 0–40 days of storage. In the 80 days of storage, the downregulated genes were enriched in ‘response of biotic and abiotic stress’. The genes enriched in ‘defense response’, ‘plant–pathogen interaction’, and ‘MAPK signaling pathway-plant’ were downregulated in the early periods and upregulated in late periods. This suggested that 8N035 responded to stress at the early stage of storage, while 8N258 responded to stress at the late stage of storage.

In the Group 35/35 (Fig. 6C, D, Table S8, S9) and Group 258/258 (Fig. 6E, F, Table S10, S11), some plant hormone binding and response functions were enriched. In the early stage of storage, the upregulated genes were enriched in ‘abscisic acid binding’ in both 8N035 and 8N258 and ‘ethylene binding’ in 8N035. The downregulated genes were enriched in ‘response of gibberellin’ in 8N258. Abscisic acid, gibberellin, and ethylene regulate the seed dormancy. This result indicated that the plant hormones regulated the dormancy during the early storage stage in 8N035 and 8N258. The downregulation of the genes involved in gibberellin response might contribute to the breaking of dormancy in the early storage stage, which cause the bud began to sprout in 8N258. In addition, the genes involved in ‘response of amino acid transport and export’ was upregulated in 8N258 during 0–40 days of storage, which might be related to the dormancy release and the bud sprout.

### Co-expression network analysis and key regulatory gene identification

**Fig. 7:**
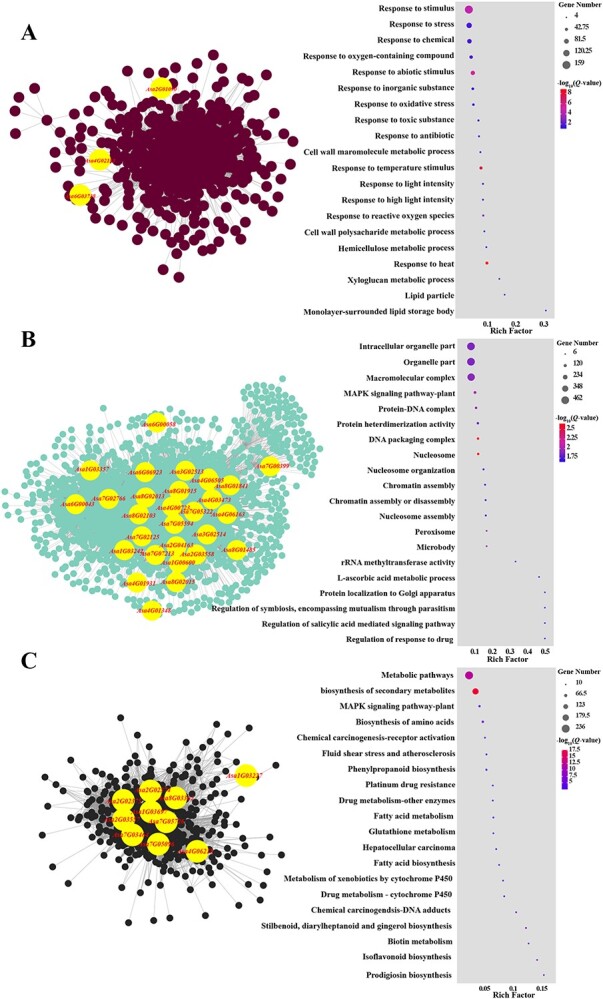
Three key modules (brown, turquoise, and black) identified from co-expression network and GO enrichment constructed using DEGs in the brown module **(A),** the turquoise module **(B),** and black module **(C)**. The genes associated with storability from GWAS results are marked with yellow in the co-expression network.

To identify the key regulatory genes involved in storability, weighted gene correlation network analysis (WGCNA) was performed using the fragments per kilobase of transcript sequence per millions (FPKM) of the DEGs. Among the identified 26 modules, three important modules (brown, turquoise, and black) based on gene expression pattern were chosen for enrichment analysis and key regulatory gene screening. In the brown module, 613 DEGs with higher expression levels in the 8N035 than the 8N258 accession at 80 and 120 days after storage were mainly enriched in ‘response to stresses’ and ‘cell wall metabolic process’ (Fig. 7A, Fig. S4). In the turquoise module, 3,698 DEGs with different expression levels in the 8N035 accession and the 8N258 accession during the entire storage process were mainly enriched in ‘genetic material’ and ‘organelle’ (Fig. 7B, Fig. S4). In the black module, 812 DEGs with higher expression levels in the 8N035 than the 8N258 accession at 40 days after storage were mainly enriched in ‘prodigiosin, isoflavonoid, biotin, and other antioxidant substance biosynthesis and metabolism’ and ‘biosynthesis of secondary metabolites’. In these modules, the genes with top 10% degree were selected as hub genes, including 61 hub genes in the brown module, 396 genes in the turquoise module, and 74 hub genes in the black module (Table S12, S13, S14).

Combined with GWAS, three associated genes were identified in the brown module, 28 associated genes were identified in the turquoise module, and six associated genes were identified in the black module (Fig. 7, Table S12, S13, and S14). The genes were associated in GWAS and also selected as hub genes were identified as the key regulatory genes, including *Asa6G00043* in the turquoise module, *Asa2G02374* and *Asa7G05726* in the black module. Notably, *Asa6G00043*, which was associated with RBC, was annotated as *LLA-115*, which could be exogenously induced by GA and might be related to the dormancy process [38]. *Asa2G02374*, which was associated with DR, was annotated as *alliinase* gene. Alliinase plays a critical role in the plant defense mechanism [39]. *Asa7G05726*, which was also associated with DR, was annotated as *cytochrome P450 89A2*. Cytochrome P450 is involved in the detoxification of xenobiotics [40].

### Joint analysis to identify important candidate genes

**Fig. 8:**
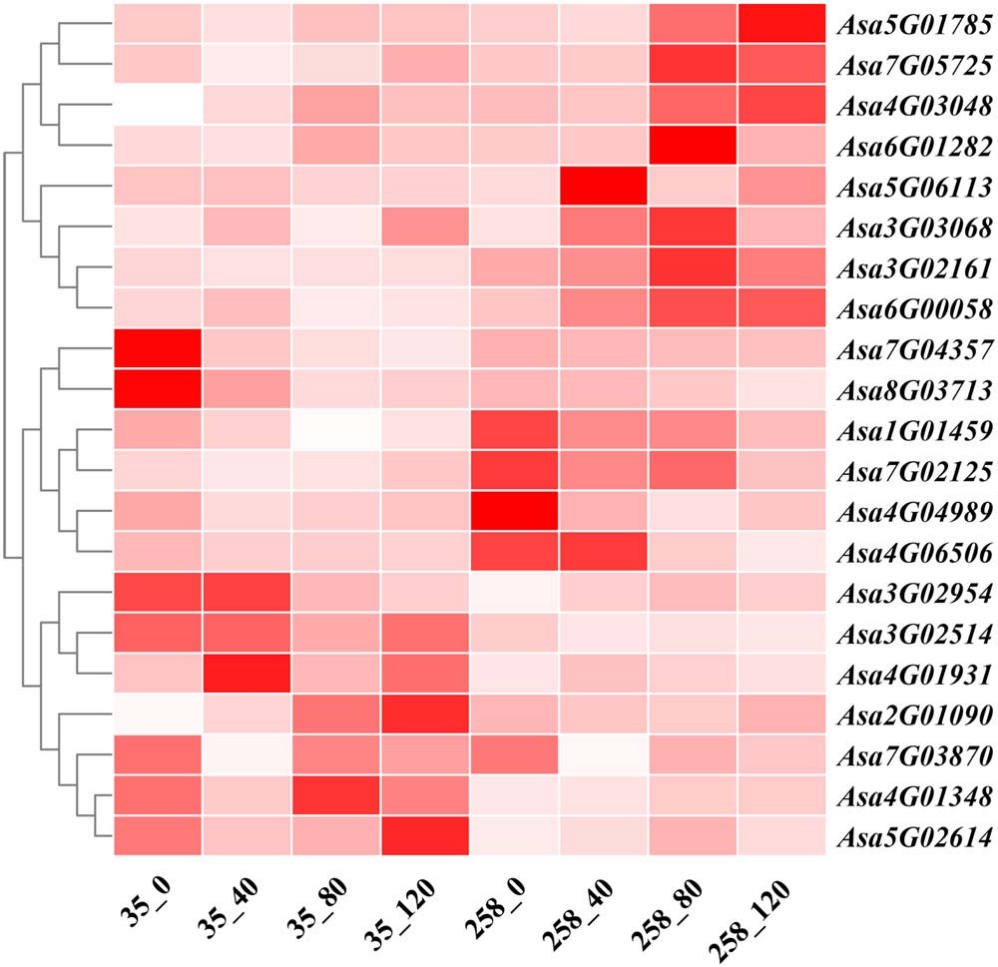
Heat map of the expression levels of important candidate genes in 8N035 and 8N258 at 0, 40, 80, and 120 days after storage

Using conjoint analysis of GWAS, selective sweep, and transcriptome, 21 important candidate genes were identified (Fig. 8). Thereinto, some of genes were involved in storage substances transport and metabolism (Table S15). *Asa5G01785* and *Asa6G00058* were annotated in carbohydrate transport and metabolism function [41, 42]. The *Asa5G01785* encoded Beta-galactosidase 2, which belongs to beta-galactosidase family members. The *Asa6G00058* encoded D-xylose-proton symporter-like 2 protein, which belongs to the sugar porter (SP) family of MFS transporters. The expression levels of these above genes were higher in 8N258 than 8N035 during storage, which coincided with the phenomenon of gradual convergence of cellular contents toward the vascular bundles for bud growth in 8N258. In addition, *Asa4G01931* encoded homeobox protein knotted-1-like 3 protein, which belongs to KNOTTED1-like homeobox (*KNOX*) transcription factor. *KNOX* plays a positive role in regulating ABA signaling [43]. The expression level of this gene is higher in 8N035 than 8N258. *Asa4G04989* encoded E3 ubiquitin-protein ligase SINAT5. The homolog of this gene promotes ubiquitin-related degradation of NAC1 to attenuate auxin signals [44]. The expression level of this gene is high in 8N258 after 0 and 40 days of storage. ABA can prolong dormancy and auxin can break dormancy in plant seeds. Therefore, it was possible that *Asa4G01931* and *Asa4G04989* controlled the dormancy process through the regulation of endogenous plant hormones, which influenced the storability in garlic.

## Discussion

Evaluation and identification of germplasm resources include phenotypic evaluation and genotype identification. Phenotypic evaluation is to determine the traits of a large number of germplasm resources for many years, so as to screenz high-quality resources for production and breeding. At present, the studies about phenotypic evaluation of garlic germplasm resources mainly measure the growth traits, quality traits, and the resistance traits [45, 46]. In this study, a total of 501 garlic accessions worldwide were used to evaluate the bulb storability. Because garlic bulbs appear yellowing, water loss, sprouting, and rot during long-term storage, four traits including DI, DR, SR, and RBC were chosen to assess the storability of garlic bulbs from a phenotypic perspective. The result demonstrated that DI was less susceptible to external conditions and was highly correlated with other traits (Fig. 1), suggesting that the DI could be identified as an all-encompassing index for evaluating the storability of garlic bulbs.

During storage, most of plants have the accumulation of ROS, which causes serious physiological disorders [47]. Against the ROS burst, garlic has unique strong antioxidant substances. In fresh garlic, alliin is the main sulfur compound, which is the main flavor source of garlic and has strong antioxidant properties. However, due to its instability, alliin is easily decomposed into allicin by alliinase with the increase of storage time, and further decomposed into organosulfur volatiles. These organosulfur volatiles also possess strong antioxidant activity [48]. In addition, flavonoids, phenolic acids, and other beneficial nutrients are also found in garlic [49]. In this study, *Asa2G02374,* annotated as alliinase gene, was found as a key regulatory gene. Alliinase breaks down allicin to produce allicin. *Asa8G03362* encoded transcription factors MYB2. These genes could provide a reference for the study of the antioxidant mechanism of garlic postharvest, improve the storage of garlic bulb, and enhance the economic value of aged garlic.

When dormancy is broken, seeds will undergo a respiratory jump due to germination. The respiratory metabolic pathway of plants requires a range of enzymes and storage substances [50, 51]. During sprouting, the substances were transported to the base of the bud through the vascular bundle [52]. In our study, the convergence of cellular contents to the vascular bundle was observed to be quite different between the SS and WS accessions. Moreover, the genes involved in storage substances transport and metabolism have been identified as candidate genes, including *Asa5G01785*, *Asa6G00058*, and *Asa2G00769*. Plant dormancy and germination are connected to plant hormones. ABA, GA_3_, and ethylene are known to be major contributors to the sprout process [53–55]. In garlic, exogenous ABA can prolong dormancy, while GA_3_ is used to relieve dormancy [56]. *Asa4G01931* as candidate gene is a member of the KNOX transcription factor, which may inhibit germination by regulating plant hormones. *Asa4G04989*, as the candidate genes, its homolog gene promotes ubiquitin-related degradation of NAC1 to attenuate auxin signals. *Asa3G02513* encoded Gibberellin 3-beta-dioxygenase. These genes might influence the storability of garlic bulbs through regulating dormancy processes controlled by plant hormones and consumption of nutrients after dormancy release. The identification of these genes provides a crucial basis for functional validation and the development of new storability varieties through transgenic or gene editing techniques.

Garlic rot is usually caused by the attack of outside pathogens, with FOC being the most destructive. In garlic, several studies have been conducted on resistance genes, for instance, *PR*, *TLP*, *DREB*, and *SWEET* family genes and miRNAs [18–20, 57, 58]. In our research, some genes were involved in the resistance. For example, *Asa2G00638* and *Asa8G03714* have been found to be related to drug resistance. The former encoded DETOXIFICATION protein, and the latter encoded an ABC transporter G family member. Detoxification proteins are a class of proteins for degradation or elimination of endogenous and exogenous toxins or medicines [32]. ABC transporters G family members have been demonstrated to take part in pathogen response, diffusion barrier formation, or plant hormone transport in *Arabidopsis* [59]. Enhancing the disease resistance of garlic could be a major step toward reducing the issue of garlic rotting. Therefore, the identification of resistance genes had great significance for the improvement of garlic storability. These key storage-related candidate genes could be chosen for accelerating the process of molecular breeding of garlic.

## Materials and methods

### Plant materials

A total of 501 garlic accessions from 38 countries (Table S1) were planted in two consecutive years (2021 and 2022) at the experimental field of the Vegetable Research Center of International Agricultural High and New Technology Industrial Park, Chinese Academy of Agricultural Sciences. The fresh bulbs were harvested in June of the following year. After natural drying, the 30 bulbs of each garlic accession were stored in a warehouse, with three biological replicates and 10 bulbs for each replicate. In 2022, one accession, 8N035, with strong storability and one accession, 8N258, with weak storability were chosen for phenotypic and cytological observations. The bulbs at 0, 40, 80, and 120 days after storage were sampled and cut into pieces, some of which were put in FAA solution, and the remaining pieces were immediately frozen in liquid nitrogen and stored at −80°C for RNA extraction.

### Storability evaluation of garlic bulbs

To determine the storability of the 501 garlic accessions, four traits including DI, DR, SR, and RBC were measured with garlic descriptors and data standards that were developed through the International Plant Genetic Resources Institute [60]. The DI was calculated using the equation DI = ∑(s_i_n_i_)/9 N × 100%. In this equation, s_i_ represented the decay classification, which could be found in Table S16, n_i_ represented the number of bulbs in each decay classification, and N represented the number of all bulbs in one accession. The DR was the number of decaying cloves as a percentage of the total number of cloves in each bulb. The SR was the number of sprouted cloves as a percentage of the total number of cloves in each bulb. The RBC was the ratio of length of bud and clove. These phenotypic data were statistically analyzed in SPSS software (version 20.0.0). The result was then visualized using GraphPad prism (version 9.3.0). The lme4 package in R (version 4.3.1) software [26] was used to estimate the broad-sense heritability (*H^2^)* with the formula *H^2^* = δ^2^g/(δ^2^g + δ^2^ge/n + δ^2^/nr), where δ^2^g was the genotypic variance, δ^2^ge/n was the genotype × environment interaction variance, δ^2^n was the number of environments, and r was the number of replicates for each environment. BLUP was also calculated using the lme4 package [26].

### Genome-wide association analysis of storability-related traits

The 2022 data, 2023 data, two-year average, and BLUP values of four storability-related traits (DI, DR, SR, and RBC) were performed GWAS with the MLM model in GEMMA software (version 0.98.1) [61] using the high-quality SNPs that were obtained from the simplified genome-sequencing data in previous study [28]. The MLM in GEMMA software was used for GWAS using the equation y = *Xα* + *Sβ* + *Kμ* + *e*. The *Xα* was the SNP genotype, acting as a marker effect; *Sβ* was the structural *Sn* matrix, acting as a fixed effect; *Kμ* was the relative kinship matrix, acting as a random effect; *e* was the residual.

The genetic structure and estimated admixture proportions were analyzed by the ADMIXTURE (version 1.3.0) [62]. PCA analysis was conducted to evaluate genetic structure using the GCTA software (version1.93.2) [63].

The first three principal components and population structure (Fig. S5, Tables S17, S18) were used for correction in GWAS. The determination of threshold was identified by Bonferroni correction. The effective number of independent SNPs was 1,378,585, and the *P*-value threshold for significance was 7.26 × 10^−7^ (1/1,378,585), which corresponded to a -log_10_ (*P*-value) of 6.13 [64].

### Cytological observation through paraffin sections

The widest part of garlic bulbs with SS (8N035, 8N239, 8N435, 8N1005, 8N1162, 8N1672) accessions and WS (8N151, 8N234, 8N258, 8N314, 8N402, 8N503) accessions were stored in FAA solution to visualize the cell morphology. The samples were dehydrated using gradient of alcohol concentrations (75%, 85%, 95%, and 100%), and xylene was used to make the material transparent. Subsequently, the samples were embedded in paraffin and cut into sections with a paraffin microtome. The sections were stained with Safranine O-Fast Green Stain to differentiate between ductal cells and parenchyma cells. Finally, the image processing and quantification were conducted using the 3DHISTECH software (Budapest, Hungary).

### Transcriptome analysis

Total RNAs of garlic bulbs of the 8N035 and 8N258 accessions were extracted using the Quick RNA Isolation Kit (Huayueyang, China). The quantity of the extracted RNA was assessed through NanoDrop 2000 (Thermo Scientific, Waltham, MA, USA) and Bioanalyzer 2100 System (Agilent Technologies, Santa Clara, CA, USA). The RNA-seq was conducted on the Novaseq-PE150 platform. After quality control and filtration, Hisat2 (version 2.0.5) software was utilized for mapping the clear reads to the *A. sativum* genome. The expression level of each gene was calculated as fragments per kilobase of transcript sequence per millions (FPKM). Genes showing |Log_2_FoldChange| ≥ 1 and *Q*-value ≤.05 were identified as DEGs. Gene ontology enrichment of these DEGs was performed using Blast2GO. Metabolic pathways were analyzed using the KEGG program. When the *Q*-value ≤.05, GO terms and KEGG pathway were defined as significantly enriched.

### Co-expression network analysis

By utilizing WGCNA (version 1.70–3) in R software, a co-expression network of the DEGs during garlic storage was created, with a threshold of 0.3. The key modules were chosen by eigengene expression (Fig. S4). Connections with weights >0.2 were identified and represented using the Cytoscape software (version 3.10.0) [65]. The top 10% degree of module genes were selected as hub genes.

### Selective sweep analysis and candidate genes identification for garlic storability

To identify potential regions of selection involved in the process of storage, The accessions in SS group and accessions in SD group were used to calculated π and *Fst* with VCFtools (version 0.1.14) [66], setting a sliding window of 100 kb with a step size of 10 kb. The regions with the top 1% of πSS/πSD ratio values (≥15.83) and *Fst* values (≥0.27) were selected as potential selected regions. The genes selected by GWAS, selective sweep, and transcriptome analyses were identified as the candidate genes involved in garlic storability.

## Supplementary Material

Web_Material_uhae260

## Data Availability

The raw RNA-Seq data have been deposited into the BIG Submission Genome Sequence Archive (GSA) under the project accession numbers CRA014298. The other supplementary figures and tables are summarized in the Supplementary Data files.
