# Supplementary material for: Integrated genome-wide association and transcriptomic studies reveal genetic architecture of bulb storability of plentiful garlic germplasm resources
Source: Hortic Res. 2024 Sep 16;11(12):uhae260. doi: 10.1093/hr/uhae260 (PMC11630311; doi:10.1093/hr/uhae260)
Supplement: Web_Material_uhae260 [file web_material_uhae260.zip › supplement_figure_information.docx]

**
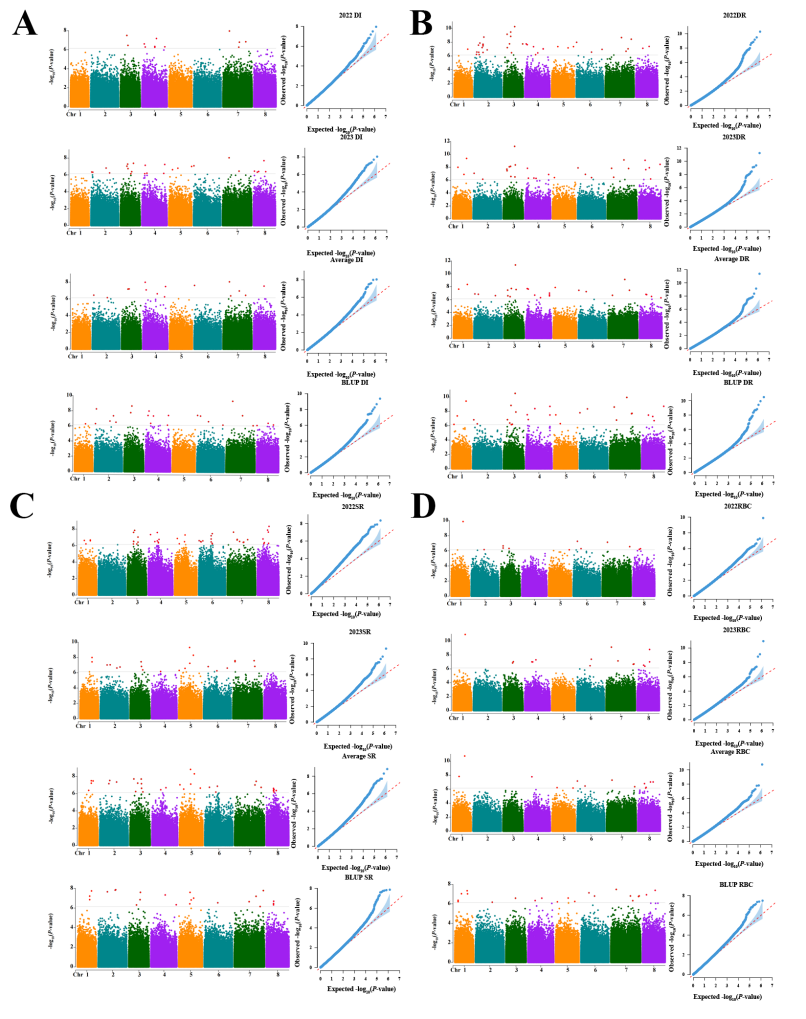
**

**Fig. S1.** Manhattan plot for the four storability- related traits including the decay index **(A)**, decay rate **(B)**, sprouting rate **(C),** and bud to clove ratio **(D).** The plots from top to bottom respectively represent the GWAS results of the 2022 data, 2023 data, two-year average, and BLUP.


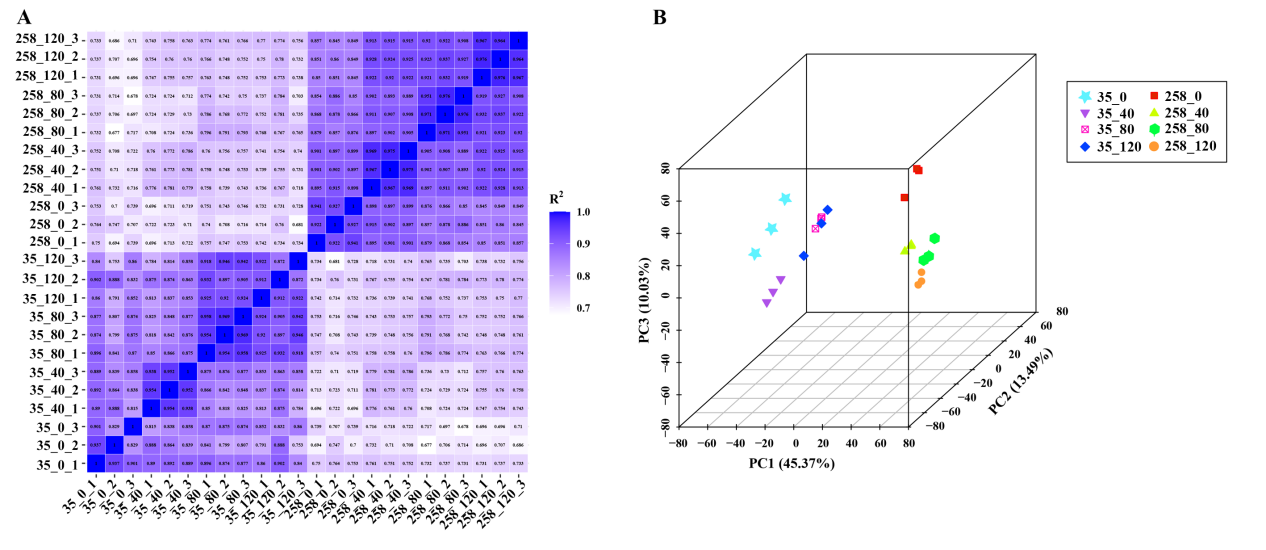


**Fig. S2.** Statistical analyisis of between RNA-seq samples. (A) Heat map Pearson correlation between samples. (B) Principal component analysis (PCA) showing the first three principal components.


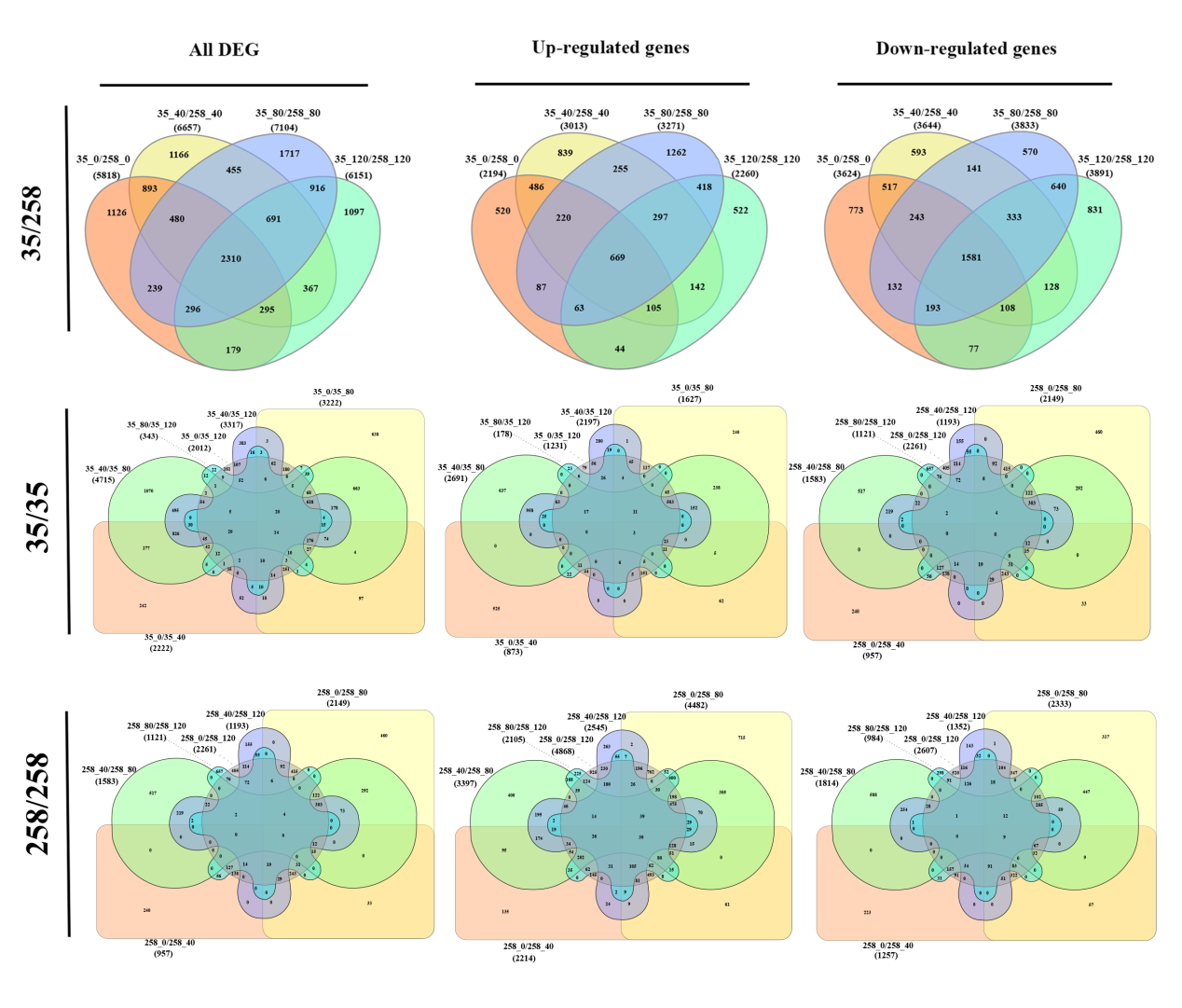


**Fig. S3.** Venn diagram of the all DEGs, up-regulated and down-regulated genes in 35/258 (35_0/258_0, 35_40/258_40, 35_80/258_80, 35_120/258_120), 35/35 (35_0/35_40, 35_0/35_80, 35_0/35_120, 35_40/35_80, 35_40/35_120, 35_80/35_120) and 258/258 (258_0/258_40, 258_0/258_80, 258_0/258_120, 258_40/258_80, 258_40/258_120, 258_80/258_120).


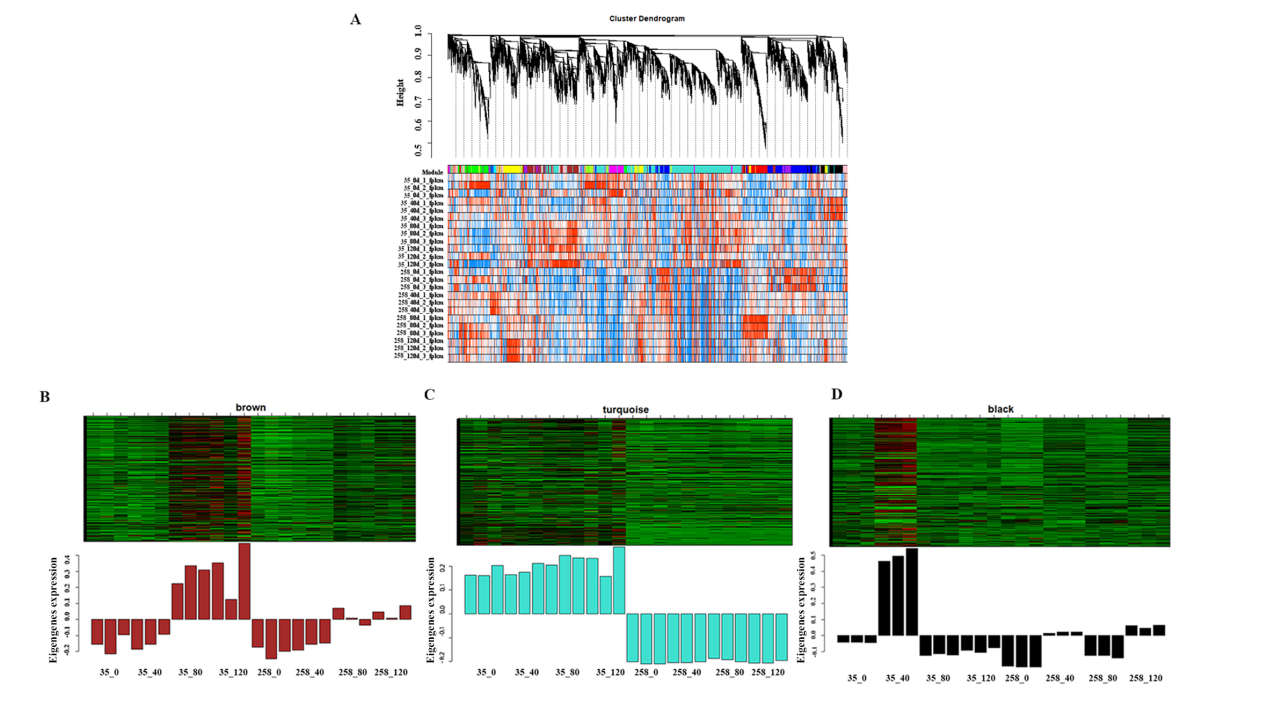


**Fig. S4.** Weighted gene co-expression network analysis (WGCNA) of differentially expressed genes (DEGs) identified from 8N035 and 8N258 in 0, 40, 80, 120 days after storage. (A) Hierarchical cluster tree and heat map showed 26 modules. (B, C, D) Bar graph and heat map represent the expression pattern in the brown (B) and turquoise (C) and black (D).

**
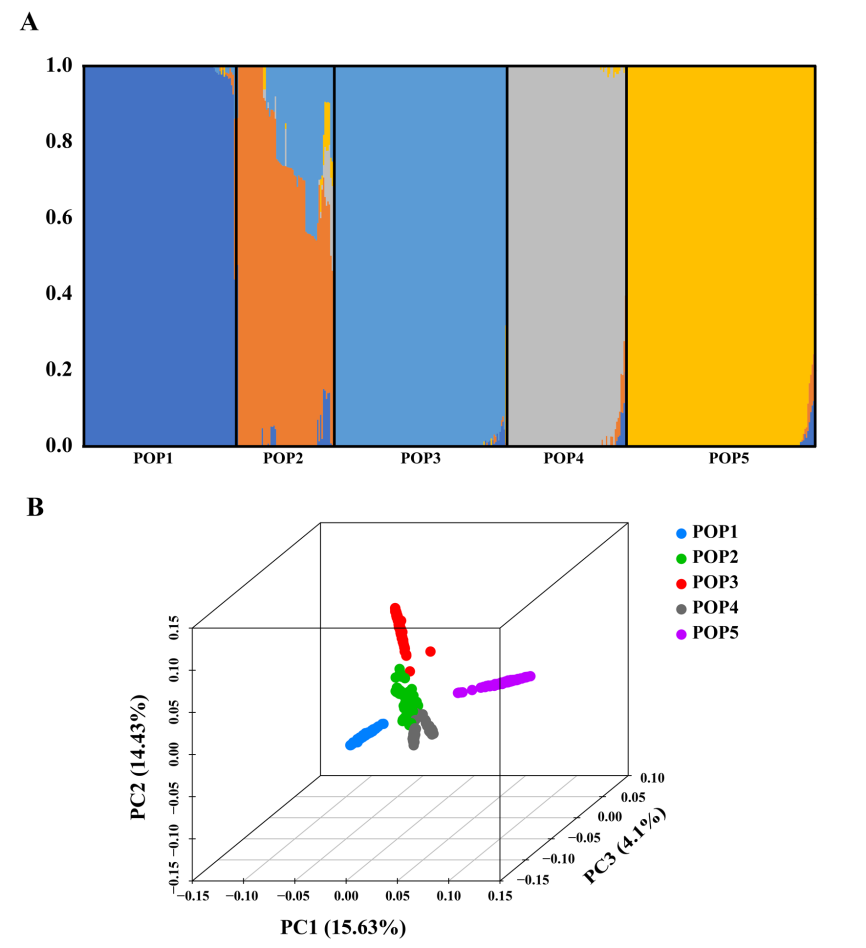
**

**Fig. S5** Population structure and phylogenetic analysis of 501 garlic accessions. (A) Population structure of the garlic accessions for K=5. (B) Principal component analysis (PCA) plots showing the first three principal components.
