# Supplementary figures and images for: Integrated genome-wide association and transcriptomic studies reveal genetic architecture of bulb storability of plentiful garlic germplasm resources
Source: Hortic Res. 2024 Sep 16;11(12):uhae260. doi: 10.1093/hr/uhae260 (PMC11630311; doi:10.1093/hr/uhae260)

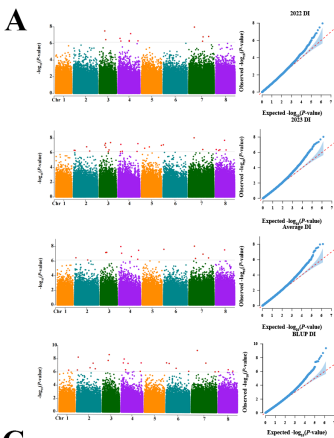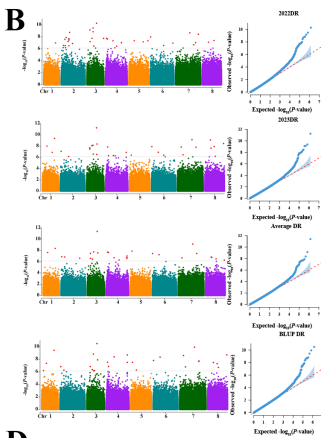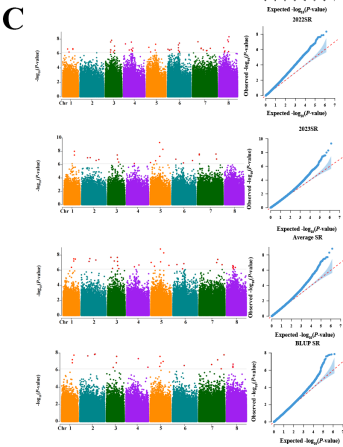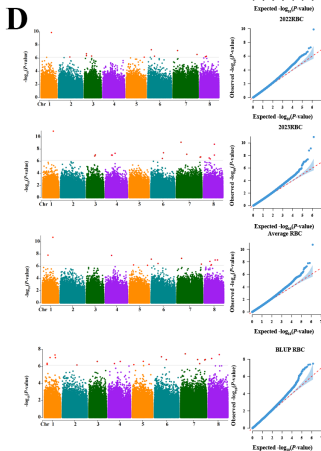

Supplement: Web_Material_uhae260 [file web_material_uhae260.zip › Figure S1.pdf]

A

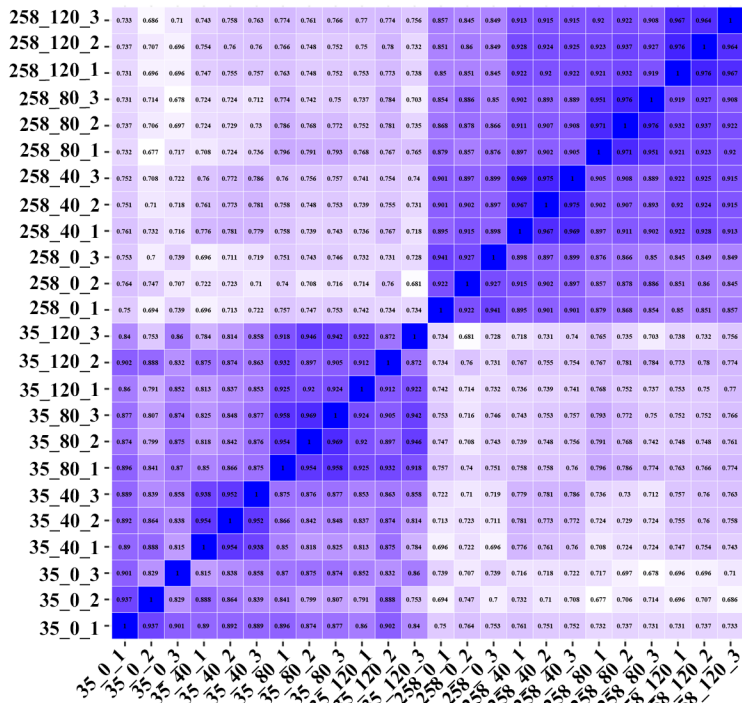 $R^2$ 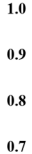

B

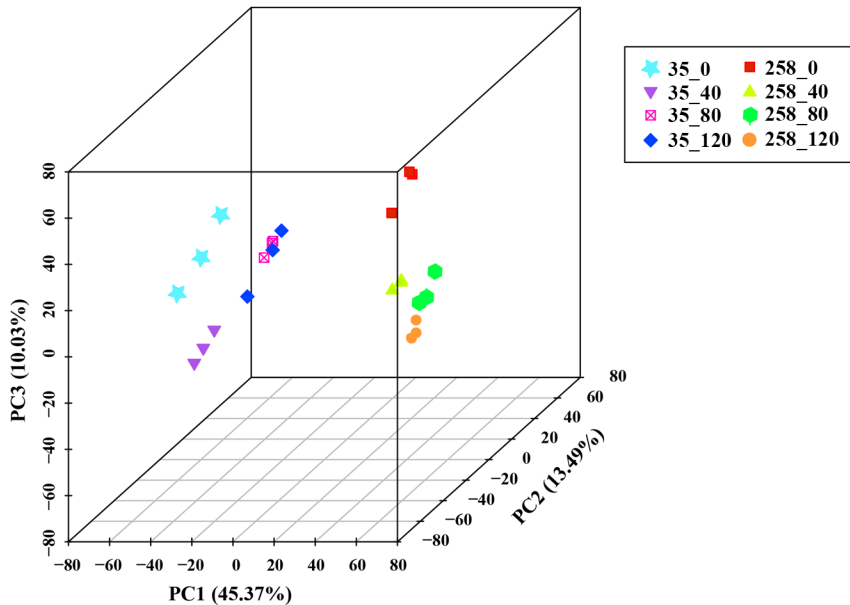

Supplement: Web_Material_uhae260 [file web_material_uhae260.zip › Figure S2.pdf]

## All DEG

## Up-regulated genes

## Down-regulated genes

35/258

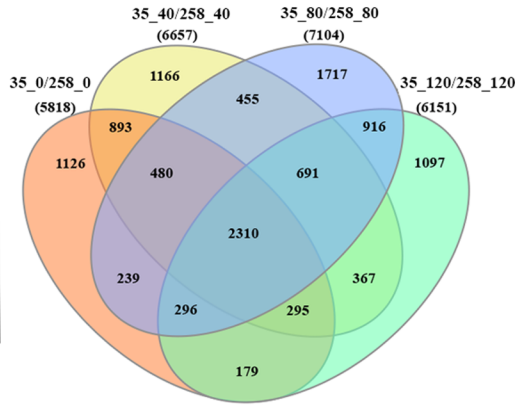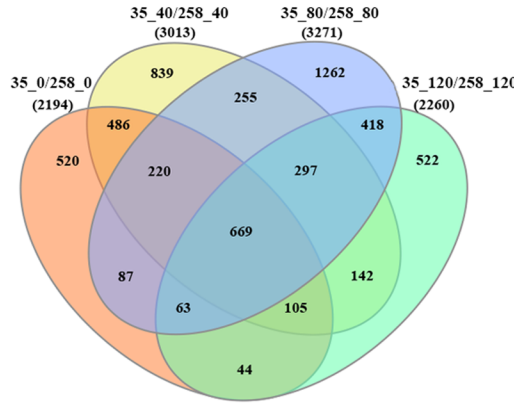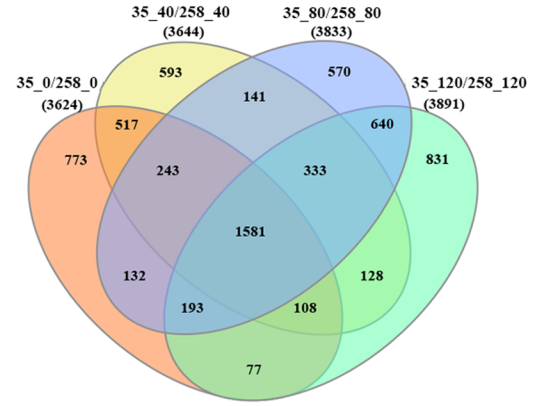

35/35

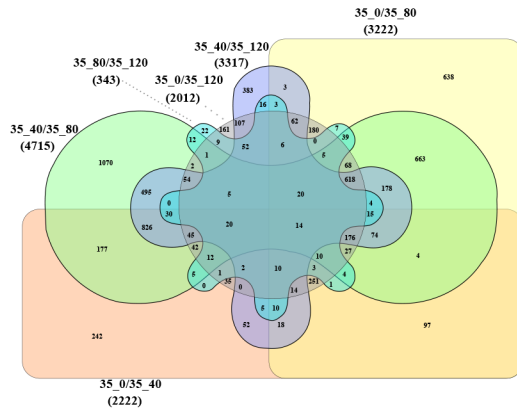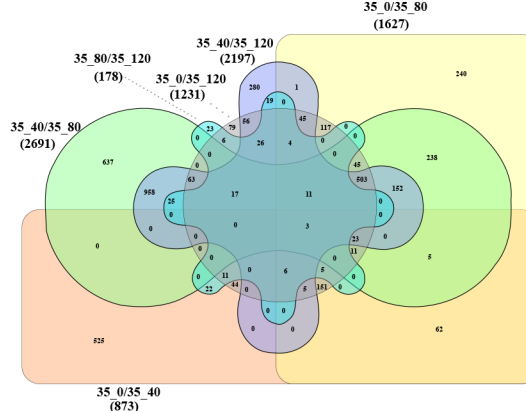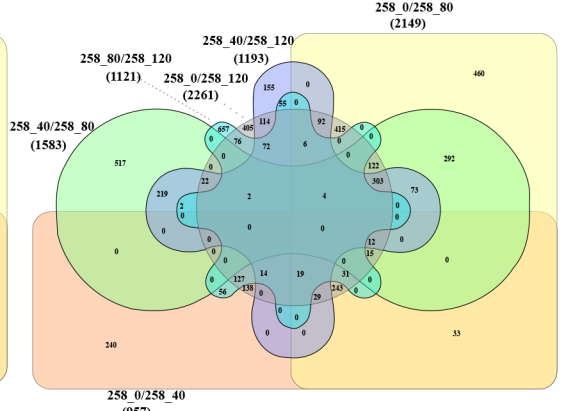

258/258

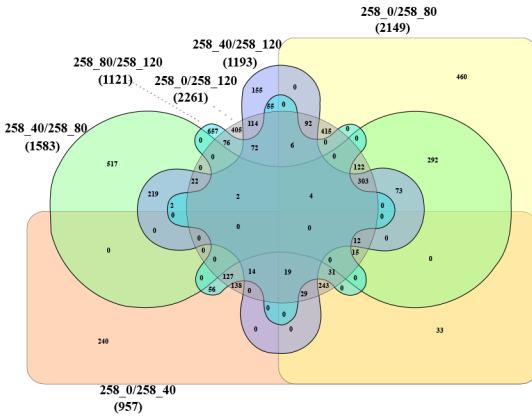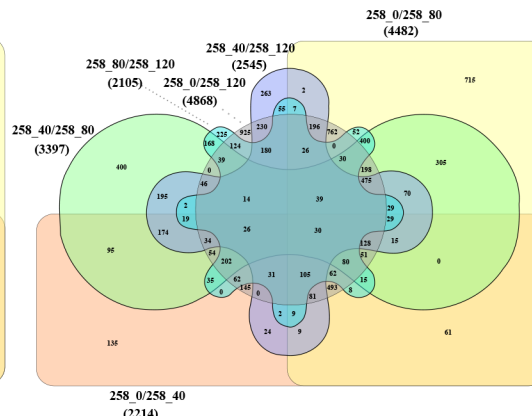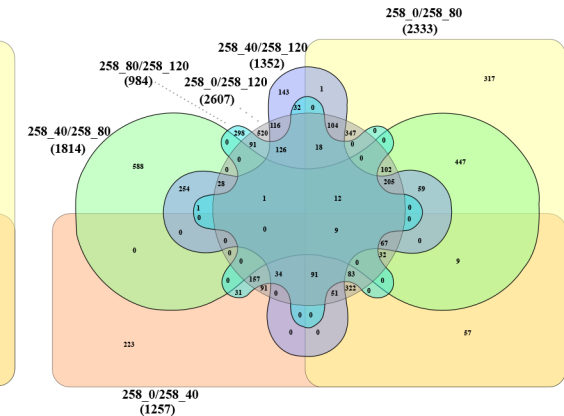

Supplement: Web_Material_uhae260 [file web_material_uhae260.zip › Figure S3.pdf]

A

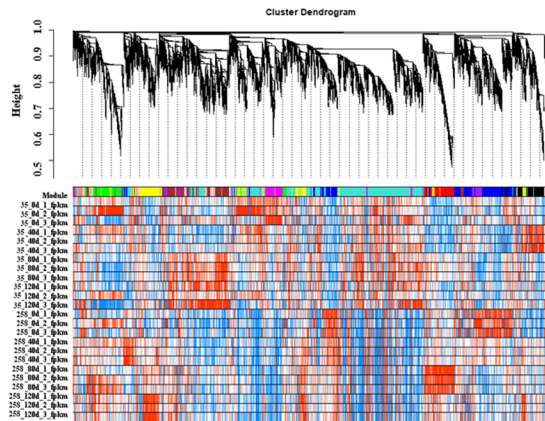**B**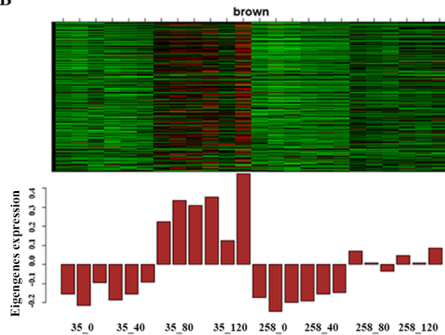

**C**

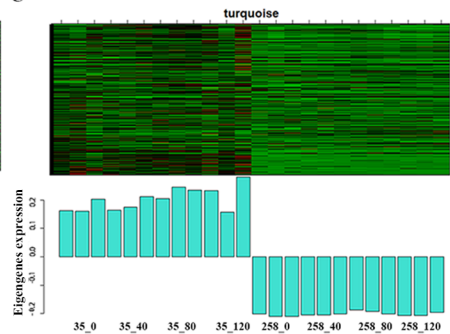

D

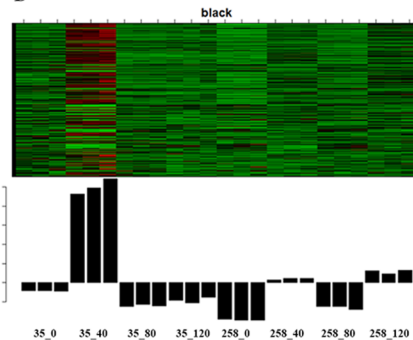

Supplement: Web_Material_uhae260 [file web_material_uhae260.zip › Figure S4.pdf]

**A**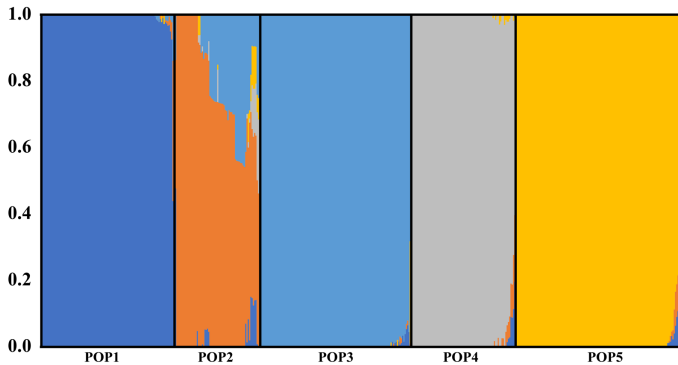**B**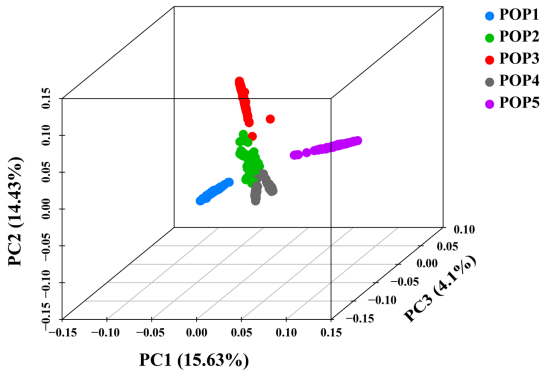

Supplement: Web_Material_uhae260 [file web_material_uhae260.zip › Figure S5.pdf]
